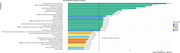# Genetic Links Between Hydrogen Sulfide Pathways and Risk of Alzheimer's Disease and Related Risk Factors: A Phenome‐Wide Approach

**DOI:** 10.1002/alz70855_107787

**Published:** 2025-12-24

**Authors:** James J.R. Brady, William R. Reay

**Affiliations:** ^1^ Wicking Dementia Research and Education Centre, University of Tasmania, Hobart, TAS, Australia; ^2^ Menzies Institute for Medical Research, Hobart, TAS, Australia

## Abstract

**Background:**

Hydrogen sulfide (H_2_S) is a promising therapeutic drug target for various pathologies, including Alzheimer's disease (AD) and related dementias (ADRD). The newly recognized gasotransmitter freely permeates cellular membranes and is vital to maintaining homeostasis, primarily via smooth muscle relaxation and vasodilatory mechanisms. Successful drug development is more likely for targets with genetic support, yet such evidence is limited for H_2_S. Here, we investigate the effect of genetic variants in H_2_S‐related pathways on thousands of phenotypes within large‐scale biobanks.

**Method:**

We conducted hypothesis‐free association tests of eight genes involved in H_2_S synthesis and metabolism with >2,400 clinical endpoints in the FinnGen biobank (N_Participants_=453,733); termed a phenome‐wide association study (PheWAS). We selected single nucleotide polymorphisms (SNPs) in these genes associated with gene expression to estimate the effect of genetically predicted gene expression on outcome phenotypes using the Wald ratio estimator. Conventional phenome‐wide association threshold‐of‐significance was applied (*p* <1x10^‐5^), and most‐significant single‐gene phenotypes screened for wider polygenic enrichment of genetic risk in sets of H_2_S‐related genes, relative to overall genome, using the MAGMA software package.

**Result:**

Analyses revealed associations between H_2_S‐related SNPs and ADRD‐related endpoints in FinnGen that surpassed multiple testing correction. Significant associations included SNP‐predicted *ETHE1* expression, associated with intra‐mitochondrial H_2_S clearance, with AD, as well cardiovascular disease endpoints. SNP‐predicted SUOX expression, involved in the final clearance of sulfur‐containing amino acids, was significantly associated with ADRD risks including type 1 diabetes, diabetic retinopathy, plus other metabolic and auto‐immune disorders. Considering more polygenic effects, we found a nominally significant enrichment of genetic risk for cerebrovascular disease, an ADRD risk, in H_2_S‐related synthesis and persulfidation pathway gene sets. This approach also revealed some evidence of enrichment of AD genetic risk in a set of proximal genetic H_2_S modifiers.

**Conclusion:**

This study is the first to provide rigorous, systematic support for the targeted investigation of H_2_S‐related genes (*ETHE1, SUOX*) and H_2_S‐related pathways for therapeutic intervention of AD and ADRD‐related risk conditions. Our hypothesis‐free outcomes lay the groundwork for future investigations of H_2_S as a modifier of neurocognitive and cerebrovascular pathology, demonstrating the utility of network‐defined gene set analyses for revealing system‐specific effects.